# SIRT4 functions as a tumor suppressor during prostate cancer by inducing apoptosis and inhibiting glutamine metabolism

**DOI:** 10.1038/s41598-022-16610-8

**Published:** 2022-07-16

**Authors:** Guohao Cai, Zhuhui Ge, Yunqiu Xu, Liangliang Cai, Pingliang Sun, Guoyu Huang

**Affiliations:** 1 Department of Anorectal Surgery, Hainan General Hospital, Haikou, China; 2 Department of Pediatrics, First Hospital of Ninghai County, Ningbo, China; 3 Department of Cardio-Thoracic Surgery, Rui’an People’s Hospital, Wenzhou, China; 4 Department of Anorectal Surgery, The First Affiliated Hospital, Guangxi University of Traditional Chinese Medicine, Nanning, China

**Keywords:** Cancer, Biomarkers

## Abstract

Localized in the mitochondria, SIRT4 is a nicotinamide adenine dinucleotide (NAD +) -dependent adenosine diphosphate (ADP) -ribosyltransferase and is one of the least characterized members of the sirtuin family. Although it is well known that it shows deacetylase activity for energy metabolism, little is understood about its function in tumorigenesis. Recent research suggests that SIRT4 may work as both a tumor suppressor gene and an oncogene. However, the clinical significance of SIRT4 in prostate cancer remains unknown. In this study, we evaluated SIRT4 protein levels in cancerous prostate tissue and corresponding non-tumor prostate tissue via immunohistochemical staining on a tissue microarray including tissues from 89 prostate cancer patients. The association between SIRT4 expression and Gleason score was also determined. Further, shSIRT4 or stable prostate cancer cell lines (22RV1) overexpressing SIRT4 were constructed via lentiviral infection. Using Cell-Counting Kit-8 (CCK-8) assay, wound healing assay, migration, and invasion and apoptosis assays, the effects of SIRT4 on the migration, invasion ability, and proliferation of prostate cancer cells were investigated. We also determined the effect of SIRT4 on glutamine metabolism in 22RV1 cells. We found the protein levels of SIRT4 in prostate cancer tissues were significantly lower than those in their non-neoplastic tissue counterparts (*P* < 0.01); a lower SIRT4 level was also significantly associated with a higher Gleason score (*P* < 0.01). SIRT4 suppressed the migration, invasion capabilities, and proliferation of prostate cancer cells and induced cellular apoptosis. Furthermore, the invasion and migration of 22RV1 cells were mechanistically inhibited by SIRT4 via glutamine metabolism inhibition. In conclusion, the present study’s findings showed that SIRT4 protein levels are significantly associated with the Gleason score in patients with prostate cancer, and SIRT4 exerts a tumor-suppressive effect on prostate cancer cells by inhibiting glutamine metabolism. Thus, SIRT4 may serve as a potential novel therapeutic target for prostate cancer.

## Introduction

Prostate cancer is the most common type of cancer diagnosed in the male population and the second leading cause of cancer-related deaths among males in the United States and other western countries^1^. According to the American Cancer Society, approximately 160,000 new cases of prostate cancer were reported in the United States in 2017, of which 32,050 patients died of the disease^2^. Prostate cancer develops through a series of defined steps, including invasive carcinoma, prostate intraepithelial neoplasia (PIN), and hormone-dependent or -independent metastasis. Although different stages of prostate cancer have histologically been well defined, relatively few underlying molecular mechanisms contributing to the initiation and development of prostate cancer are known.

The sirtuin (SIRT) protein family is a group of NAD^+^ dependent deacetylases and ADP ribosyltransferases. Humans encode seven sirtuin isoforms (SIRT1–SIRT7), which are involved in stress resistance, energy homeostasis, and aging^3^. SIRT4, an NAD^+^-dependent ADP-ribosyltransferases localized in the mitochondria, interacts with and represses glutamate dehydrogenase (GDH) activity via mono-ADP-ribosylation^4^, and facilitates glucose homeostasis, insulin secretion, and fatty acid oxidation^4–7^. Recent studies indicate that mitochondrial SIRT4 exhibits tumor-suppressing activities by promoting genomic stability^7–11^. Studies on glutamine metabolism regulation have also shown that SIRT4 may also function as an oncogene^12,13^. However, there are few studies about the effects of SIRT4 on prostate cancer cells. Thus, the role of SIRT4 in prostate cancer remains elusive.

In this study, we evaluated the associations between immunohistochemical SIRT4 protein expression in prostate cancer tissue and Gleason score to determine the clinical importance of SIRT4 expression in patients with prostate cancer. Moreover, we investigated the in vitro effects of SIRT4 on prostate cancer cell proliferation, migration, invasion, cell cycle, and prostate cancer cell apoptosis by inhibiting and overexpressing the SIRT4 gene in 22RV1 cells. Finally, we investigated the effect of SIRT4 on L-Glutamine metabolism and its role in inhibiting the function of prostate cancer cells.

## Materials and methods

Given the retrospective nature of the study, ethical approval was provided by the local ethics committee of the First Affiliated Hospital of Wenzhou Medical University (Wenzhou, China). It was conducted by following the principles of the Declaration of Helsinki.

### Patients and tissue samples

Tumor tissue specimens from 89 patients with prostate cancer who underwent surgery were acquired from the First Affiliated Hospital of Wenzhou Medical University between February 2011 and March 2016. The mean age of the patients was 70 years (range 55–90 years). Informed consent was obtained from all the participants. All patients were diagnosed with pathological type prostate adenocarcinoma. No patient underwent preoperative radiotherapy or chemotherapy before surgery. The patients’ Gleason scores were obtained from the American Joint Committee on Cancer (AJCC, 7th edition) data.

Tissue microarray (TMA) chips were obtained commercially (Superchip Inc., Shanghai, China). The TMA contained 89 prostate cancer patient specimens including tumor tissue samples and their corresponding adjacent non-neoplastic tissue specimens. A tissue cylinder (diameter, 2 mm) was stamped out from morphologically representative areas of each donor block and transferred to a recipient paraffin block. Tissue microarray blocks were 2.0 mm in diameter, and all points were covered with paraffin wax.

### Immunohistochemical analysis

The immunohistochemical assay was performed on TMA chips. The chip was deparaffinized in xylene twice for 5 min at room temperature (RT) and then rehydrated in successively graded concentrations of ethanol at 100%, 95%, 85%, and 70%, respectively for 5 min. Antigen retrieval was performed by heating the sections at 100 °C for 30 min in citrate buffer (0.05% Tween 20 and 10 mM citrate, pH 6.0) at 170 kPa at 120 °C for 5 min. Subsequently, endogenous peroxidase activity was blocked by incubation in 0.3% H_2_O_2_ in Tris–HCl buffer for 15 min at RT. The chip was then washed 3 times and incubated with a polyclonal rabbit anti-SIRT4 antibody (HPA029692, 1:400, Sigma, USA) at 4 °C in a refrigerator for more than 8 h. Subsequently, a secondary antibody was added to the chip using the GTVision Kit (Gene Tech Inc., Shanghai, China) and the chip was incubated, as per the manufacturer’s instructions. The microarray chip section was then consecutively stained with diaminobenzidine (DAB) and hematoxylin. Next, the chip was dehydrated and sealed with a coverslip. Tissues treated with only a diluent (without antibody) were used as negative controls.

Tissue wafer scanning and analysis method:

Tissue chip scanner model Pannoramic MIDI, manufacturer: 3D HISTECH (Budapest, Hungary): The tissue chip section gradually moves under the scanner lens. The moving edge is imaged, and the information on the tissue section is scanned and imaged to create a file. The file contains all the tissue information available on the tissue section. The file can be enlarged 1–400 times in the Pannoramic viewer software. Further, any part of the tissue section can be imaged. The Quant center is an analysis software program associated with the Pannoramic viewer. On scan completion, the TMA software of the Quant center analysis software can be run after setting the diameter of the wafer tissue and the number of rows and columns. The software then generates a number. The nuclei in the tissue sections that were strongly positive were colored dark brown, those moderately positive were brown yellow, those weakly positive were pale yellow, and those negative were blue using the densito quant software in Quant center. The area (unit: pixel), the percentage of positive staining, and final last histochemical score (H-SCORE) were analyzed for each tissue site. The formula for calculating the H-SCORE was as follows: H-SCORE = ∑(Pi × I) = (percentage of weakly positive cells × 1)(percentage of moderately positive cells × 2)(percentage of strongly positive cells × 3), with pi representing the percentage of the positive cells in the tissue section and i representing the intensity of the staining. The H-SCORE score ranged from 0 to 300, with a higher score indicating a stronger positive score^14,15^.

### Expression analysis of the SIRT4 Gene

We analyzed the mRNA levels of SIRT4 from 492 prostate cancer cases and 52 normal prostate tissues obtained from the TCGA database of the GEPIA website (http://gepia.cancer-pku.cn/detail.php).

### Cell lines and culture conditions

A human prostate cancer cell line, 22RV1, was procured from the Shanghai Institute of Cell Biology, Chinese Academy of Sciences, in June 2020. The cell bank used four pairs of primers, DXS52, Apo-B, MD17S5, and D2S44, to monitor the cell line variation during the passage. The cells were last tested in June 2020, and the experiment ended half a year after the cell was purchased. Cells were maintained in Dulbecco’s modified Eagle’s medium (DMEM; Gibco, USA) supplemented with 10% fetal bovine serum (Gibco, USA) and penicillin/streptomycin (Gibco, USA), and incubated at 37 °C with 5% CO_2_.

### Vector and virus production

We designed three siRNA sequences targeted to SIRT4, viz., 5ʹ-GCGTGTCTGAAACTGAATTCTʹ, 5ʹ-GCTCCTGATGGTGACGTCTTTCTCT-3ʹ and 5ʹ-GCGTTCAATGTGGAGGCCATCTGAA-3ʹ, respectively. The negative control sequence was 5′-TGTCACTCTCCGGAACGTT-3′. We purchased the lentivirus vector pHBLV-CMVIE-ZsGreen-T2A-Puro overexpressing SIRT4 and shSIRT4 from biotechnology company (Hanbio, Shanghai, China). The final titer of the lentivirus and negative control virus was 2 × 10^8^ PFU/ml. Stable overexpression of SIRT4 or shSIRT4 was achieved by transfecting 22RV1 cells with lentivirus for 72 h; colonies were isolated using puromycin for 2 weeks. Lipofectamine2000 (Thermo Fisher Science) was used as the transfection reagent.

### Reverse transcription (RT)-PCR

Total RNA from the tissues or cells was extracted using the TRIzol reagent (Invitrogen, USA). Further, 500 ng of cDNA was synthesized using a reverse transcription kit (PrimeScriptTM RT Master Mix, TaKaRa, Japan). Then, qRT-PCR was performed with thrice-diluted cDNA using the qRT-PCR Kit (SYBR® Premix Ex Taq™aqR KTaKaRa, Japan) on the DNA Engine Opticon 2 real-time detection system (BioRad). GAPDH was chosen as the internal reference gene. Primers for each gene were as follows: SIRT4 forward primer 5′- GCGAGAAACTTCGTAGGCTG -3′, reverse primer 5′- TCAGGACTTGGAAACGCTCT -3′; GAPDH forward primer 5′-TCAAGAAGGTGGTGAAGCAGG -3′, reverse primer 5′- TCAAAGGTGGAGGAGTGGGT -3′. The PCR reaction conditions were as follows: 2 min at 94 °C; followed by 40 cycles of 30 s at 94 °C, 30 s at 57 °C, and 1 min at 72 °C; and ending with 5 min at 72 °C and cooling at 4 °C. After the reaction was completed, the homogeneity of the PCR product was confirmed by analyzing the dissolution curve followed by analysis of relative gene expression by 2^-∆∆^CT.

### Western blotting

Cells were lysed with Ripa lysis buffer (Beyotime, China) supplemented with protease inhibitor cocktail (Beyotime, China). Cell lysates were separated by sodium dodecyl sulfate–polyacrylamide gel electrophoresis (SDS-PAGE) and immunoblotting. The primary antibodies utilized included rabbit anti-human SIRT4 polyclonal antibody (clone HPA029691, Sigma, USA), rabbit anti-human caspase3 (35/18 KDa) polyclonal antibody (9662, CST, USA), rabbit anti-human caspase9 (46/35 KDa) polyclonal antibody (10380-1-AP, Proteintech, China), rabbit anti-human bcl2 (30 KDa) polyclonal antibody (12789-1-ap, Proteintech, China), rabbit anti-human bax (21 KDa) polyclonal antibody (50599-2-ig, Proteintech, China), rabbit anti-human p65 (65 KDa) monoclonal antibody (8242, Cell signaling, USA), rabbit anti-human p-p65 (65 KDa) monoclonal antibody (3033, Cell signaling, USA), rabbit anti-human matrix metalloproteinase 9 (MMP9) (78 KDa) polyclonal antibody (10375-2-AP, Proteintech, China), rabbit anti-human n-cadherin (99 KDa) monoclonal antibody (14472, CST, USA), rabbit anti-human e-cadherin (135 KDa) monoclonal antibody (Ab124397, Abcam, England), goat anti-rabbit detection antibody (ab97200, Abcam, England), rabbit anti-human Lamin B1 (67 KDa) polyclonal antibody (BA1228, Boster Biological Technology, China), and rabbit anti-human glyceraldehyde 3-phosphate dehydrogenase (GAPDH) polyclonal antibody (AB-P-R 001, Goodhere, China). We cut the membranes into long strips after transferring the membranes to incubate different antibodies before incubating them to conserve antibodies. This cut membrane was placed on the development plate during the final exposure. Since we do not add the full 8 lanes each time we run electrophoresis, and we cut the membrane according to the molecular marker before incubating the antibody, our original membrane is not long enough, especially if we do not use a particularly large number of samples for each experiment. For example, when we only need to run 4 samples at a time, then the length of our membrane may be half the length of the whole membrane. This is hereby clarified.

### Biochemical assay

For the analysis of GDH activity, the biochemical activity of these cell lines was analyzed using the Human Glutamate dehydrogenase (GLDH) test kit (A123, Jiancheng Bioengineering Institute, Nanjing, China).

### Cell proliferation activity

Cells were inoculated in 96-well plates with a density of 1000 cells/well. Subsequently, 10 µL of cell counting kit-8 (CCK-8) reagent (Dojindo, Japan) was added in each well followed by culturing in a CO_2_ incubator for 2 h and absorbance determination. The final concentration of the bis-2-(5-phenylacetamide-1,2,4-thiadiazol-2-yl)ethyl sulfide (BPTES; SML0601, Sigma, USA) was 10 μmol/L. The final concentration of dimethyl α-ketoglutarate (DM-αKG; 349631, Sigma, USA) was 8 mmol/L.

### Wound healing assay

Wound healing assay was carried out to determine cell migration. Briefly, the SIRT4 overexpression, shSIRT4, and the corresponding negative virus group cells were seeded in a 6-well plate with a density of 5 × 10^5^ per well and cultured for 10 h. After this, a 200-microlite tip perpendicular to the bottom of the plate was used to design a scratch line. Subsequently, the detached cells were washed with PBS and cultured in a serum-free medium. DM-αKG at a concentration of 4 mM or 8 mM was added after scratching in the DM-αKG group. In the experimental group, cell images in the scratched area after 0 and 24 h of culture, obtained using an optical microscope, were used to calculate the distance migrated by the cells.

### Cell migration and invasion test

In the migration test, cells were suspended in a serum-free medium with a density of 3 × 10^5^ ml, and a 0.2-ml suspension was inoculated into the upper chamber of the Transwell plate (Corning Inc., Conning, New York, USA). The cells were filled with 0.6 ml culture medium containing 10% FBS. The cells were then incubated at 37 °C for 18 h. The cells in the upper compartment were then wiped with a sterile cotton swab and the membrane was soaked in 4% paraformaldehyde to immobilize the cells in the lower compartment, followed by staining with 0.1% crystal violet. The cells were counted under an optical microscope (Olympus). In the invasion test, the superior chamber was precoated with BD Matrigel™ Basement Membrane Matrigel (BD biosciences) before cell inoculation. The rest of the procedure was the same as that followed for the migration test. For DM-αKG group, the culture medium with a 4 mM or 8 mM DM-αKG was adjusted after seeding.

### Flow cytometric analysis for apoptosis rate and cell cycle

Cells were harvested by trypsinization, pelleted by centrifugation, and resuspended in PBS containing 3% fetal bovine serum. The cell apoptosis was performed with flow cytometry (C6, BD, USA) using annexin V-allophycocyanin (annexin V-APC) and 7-aminoactinomycin D (7-AAD) (BD, USA) staining. Apoptosis rate was only calculated at early apoptosis. The survival rate was calculated by cells not stained with annexin V-APC or 7-AAD. The apoptotic cells were analyzed by flow cytometry analysis using the Accuri C6 software program (BD, USA). The cell cycle was determined using a Propidium Iodide (PI)/RNase kit (BD, USA). The results were examined with the ModFit analysis software program (Verity Software House, Topsham, ME, USA).

### Cell immunofluorescence

Immunofluorescence analyses were performed as described previously^16^, using rabbit anti-human NF-κB p65 polyclonal antibody (10745-1-AP, 1:50 dilution, Proteintech Group, China), rabbit anti-human Phospho-NF-κB p65 polyclonal antibody (3033S, 1:50 dilution, CST, USA), and an Cy3-conjugated goat anti-rabbit IgG secondary antibody (BA1032, 1:100 dilution, Boster Biological Technology, China). DAPI was used for staining of nuclei and to assess gross cell morphology.

### Statistical analysis

The SPSS software package version 22.0 (SPSS, Inc., IBM, USA) was used for statistical analysis. All in vitro experiments were performed in triplicates. Data from three or more independent experiments are presented as the mean ± standard deviation. The final scores of tumor tissue and non-tumor tissue were analyzed using a paired student’s *t* test. Experiments unless specified were analyzed using the non-paired *t*-test. *P* < 0.05 (two-tailed) was considered statistically significant.

### Consent for publication

Yes.

## Results

**Figure 1:**
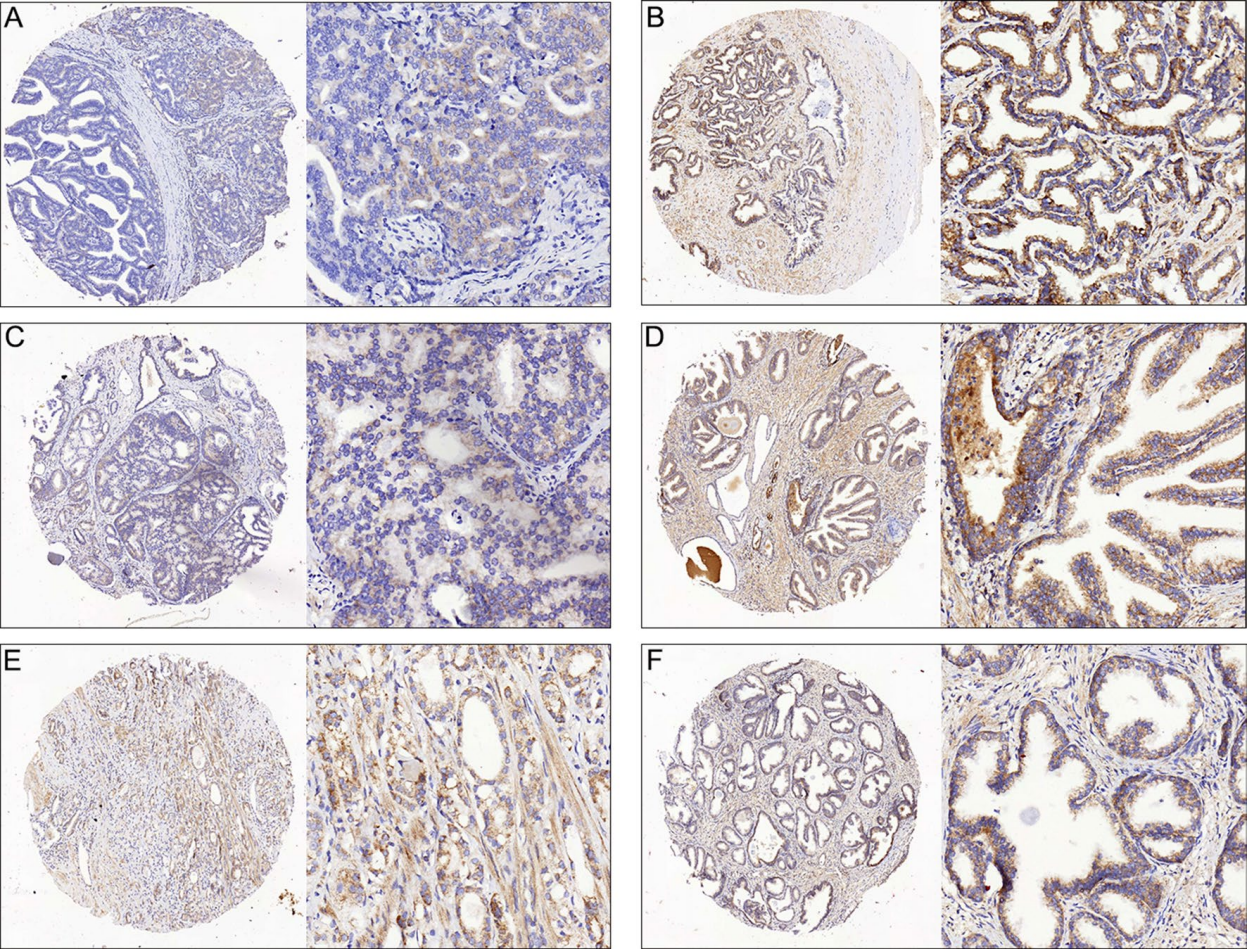
Representative immunohistochemical staining of SIRT4 in prostate tumor cells. SIRT4 is localized to the cytoplasm and is expressed at lower levels in tumor tissues as compared with adjacent non-neoplastic prostate tissues. The micrographs showed weak (**A**), median (**C**) and high (**E**) expression of SIRT4 in the prostate cancer tissues. The relevant expression of SIRT4 in corresponding adjacent non-neoplastic prostate tissuesin cases showing (**A**), (**C**) and (**E**) were shown in (**B**), (**D**) and (**F**), respectively (magnification: left panel 100 × , right panel 400 ×).

**Figure 2:**
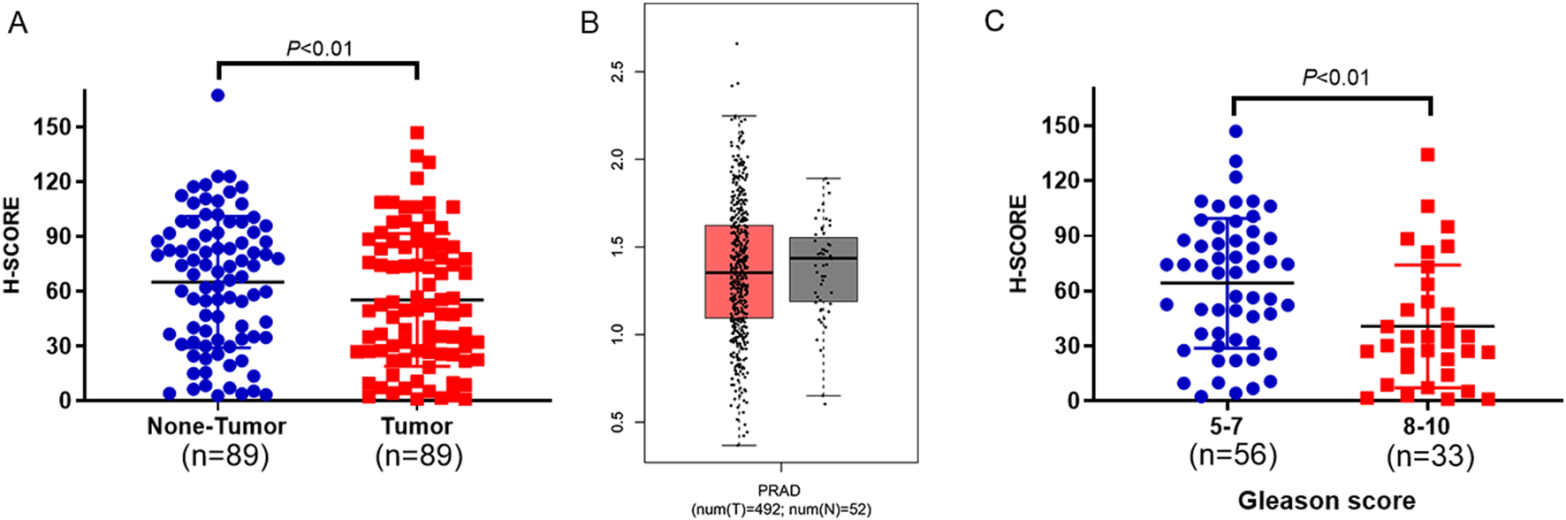
SIRT4 is downregulated in prostate cancer tissues and correlated with higher Gleason scores. (**A**) SIRT4 protein levels were measured in 89 prostate cancer tissues and paired with adjacent non-neoplastic prostate tissues using a tissue microarray. SIRT4 protein levels are lower in tumor tissues compared with adjacent non-neoplastic prostate tissues (*P* < 0.01). Histochemical scores (H-SCORE) were analyzed by Quant center version 2.2 (3D HISTECH, Budapest, Hungary). Statistical analysis was performed using the statistical software program SPSS version 22.0 (IBM Corp., Armonk, NY, USA). (**B**) TCGA data were analyzed online using the GEPIA website (http://gepia.cancer-pku.cn/detail.php). Box plots depict the reduced expression of SIRT4 in prostate cancer (n = 492) than normal prostate tissue (n = 52) using bioinformatics analysis. (**C**) SIRT4 expression is lower in prostate patients with a higher Gleason score (*P* < 0.01). The boxes represent the interquartile range, whiskers represent the 5th-95th percentile range, and bars represent the median.

SIRT4 is downregulated on immunohistochemical staining of human prostate cancer and adjacent non-neoplastic tissues. We found that SIRT4 was predominantly localized in the cytoplasm. The immunostaining intensity of SIRT4 in the tumor tissue was notably lower (Fig. 1) than in the adjacent non-neoplastic prostate tissues. The difference was statistically significant (Fig. 2A). We further analyzed the difference in RNA expression of SIRT4 in 492 prostate cancers and 52 normal prostate tissues through the GEPIA website. The results were consistent with our immunohistochemical findings that SIRT4 RNA levels were lower in prostate cancers than in normal prostate tissues (Fig. 2B).

### The association of SIRT4 immunostaining intensity with Gleason score in prostate cancer

To determine the clinical significance of SIRT4 expression in patients with prostate cancer, the relationship between SIRT4 expression and the Gleason score in prostate cancer was analyzed. A significant negative correlation was observed between SIRT4 expression and the Gleason score in prostate cancer in the present study (Fig. 2C). We found that a higher Gleason score was exhibited in patients with low expressions of SIRT4.

### Effect of SIRT4 on proliferation, migration, invasion capabilities, and cell cycle of prostate cancer cell lines

**Figure 3:**
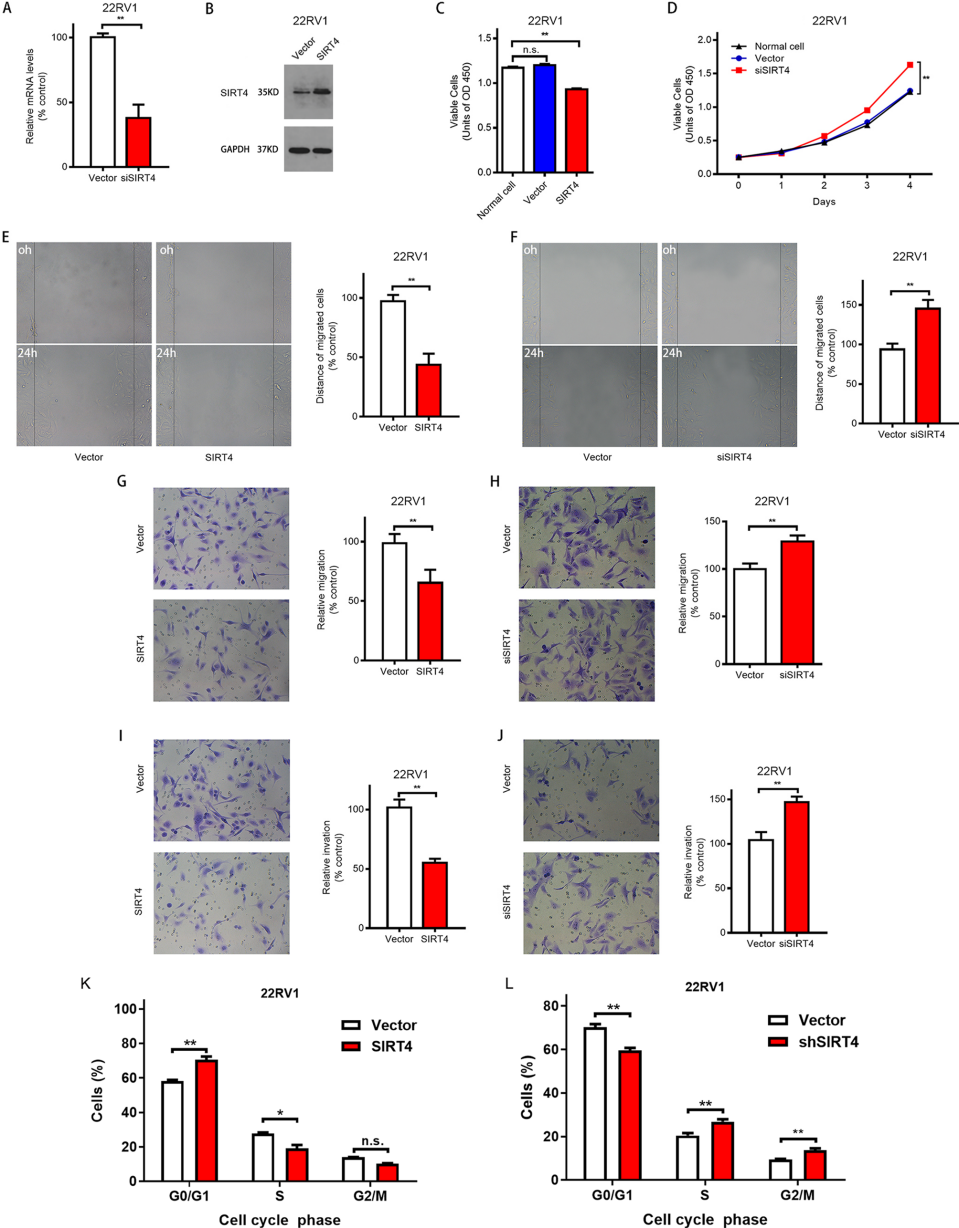
SIRT4 inhibits cell proliferation, migration, invasion capabilities, and cell cycle of the prostate cancer cell. (**A**) 22RV1 cells transfected with vector or shSIRT4 are analyzed with qRT–PCR to determine SIRT4 expression. (**B**) Western blot analysis of SIRT4 overexpression following treatment with puromycin 2 μg/ml for 2 weeks (right), GAPDH as an internal control. The original blots are represented in Supplementary Fig. 1. (**C**) The proliferation of vector and SIRT4-OE 22RV1 cells. The cell Proliferative activity is measured at 72 h after seeding. (**D**) Proliferation curve of vector and shSIRT4 22RV1 cells. The cell Proliferative activity is measured every 24 h for four consecutive days. (**E**) A representative picture of the wound healing assay of vector and SIRT4-OE 22RV1 cells (left). The ratio of cell migration in SIRT4-OE cells to that of vector cells (right). (**F**) A representative picture of the wound healing assay of vector and shSIRT4 22RV1 cells (left). The ratio of cell migration in SIRT4-OE cells to that of vector cells (right). (G and I) The migration (**G**) and invasion (**I**) of 22RV1 cells as determined using transwell assays. Vector and SIRT4-OE 22RV1 cells are subjected to transwell assays. After culturing for 18 h in transwell plates, the migrated or invaded cells were stained and observed for five random fields under a 400 × microscope and counted. (**H**,**J**) The migration (**H**) and invasion (**J**) of 22RV1 cells as determined through transwell assays. Vector and shSIRT4 22RV1 cells are subjected to transwell assays. After culturing for 18 h in transwell plates, the migrated or invaded cells were stained and observed in five random fields under a 400 × microscope and counted. (**K**,**J**) The effect of overexpression or interference of SIRT4 on the cell cycle distribution of 22RV1 cells was analyzed by PI Cell Cycle Assay Kit. Modfit (Verity Software House, Topsham, ME, USA) analyzed the results, representing three independent experiments and expressed as mean ± sD. *P < 0.05, **P < 0.01.

Through pre-experiment, the interference effect of siRNA sequence 5ʹ-GCGTTCAATGTGGAGGCCATCTGAA-3ʹ was found to be the best (data not shown). We constructed stable strains with SIRT4 overexpression and suppression of 22RV1 prostate cancer cells via lentiviral infection and confirmed the results by RT-PCR (Fig. 3A) and Western Blotting (Fig. 3B). Cell proliferation assay revealed that SIRT4 overexpression significantly suppressed the proliferation of 22RV1 cells (Fig. 3C); in contrast, after SIRT4 inhibition, the proliferative activity of 22RV1 cells was significantly higher than that of the untransfected cells and negative controls (Fig. 3D). Moreover, cell migration assay showed that SIRT4 overexpression significantly lowered the wound-healing rate (Fig. 3E); the wound-healing rate, however, was accelerated on SIRT4 knockdown (Fig. 3F). Next, we found that invasion and migration abilities of 22RV1 cells were significantly reduced with SIRT4 overexpression (Fig. 3G,I), and significantly increased after SIRT4 inhibition (Fig. 3H,J). Next, we examined the effect of SIRT4 on the cell cycle of prostate cancer cells 22RV1. The overexpression of SIRT4 significantly enhanced the proportion of cells in G0/G1 phase. Moreover, it decreased the proportion of cells in the S phase than the negative control (Fig. 3K), while interference with SIRT4 provided the opposite result (Fig. 3L). This suggests that overexpression of SIRT4 induced cessation of the G0/G1 phase. Therefore, these results indicated that SIRT4 is critical in determining the proliferation, migration, invasion abilities, and cell cycle of prostate cancer cells during in vitro experiments.

### Overexpression of SIRT4 induces prostate cancer cell apoptosis

**Figure 4:**
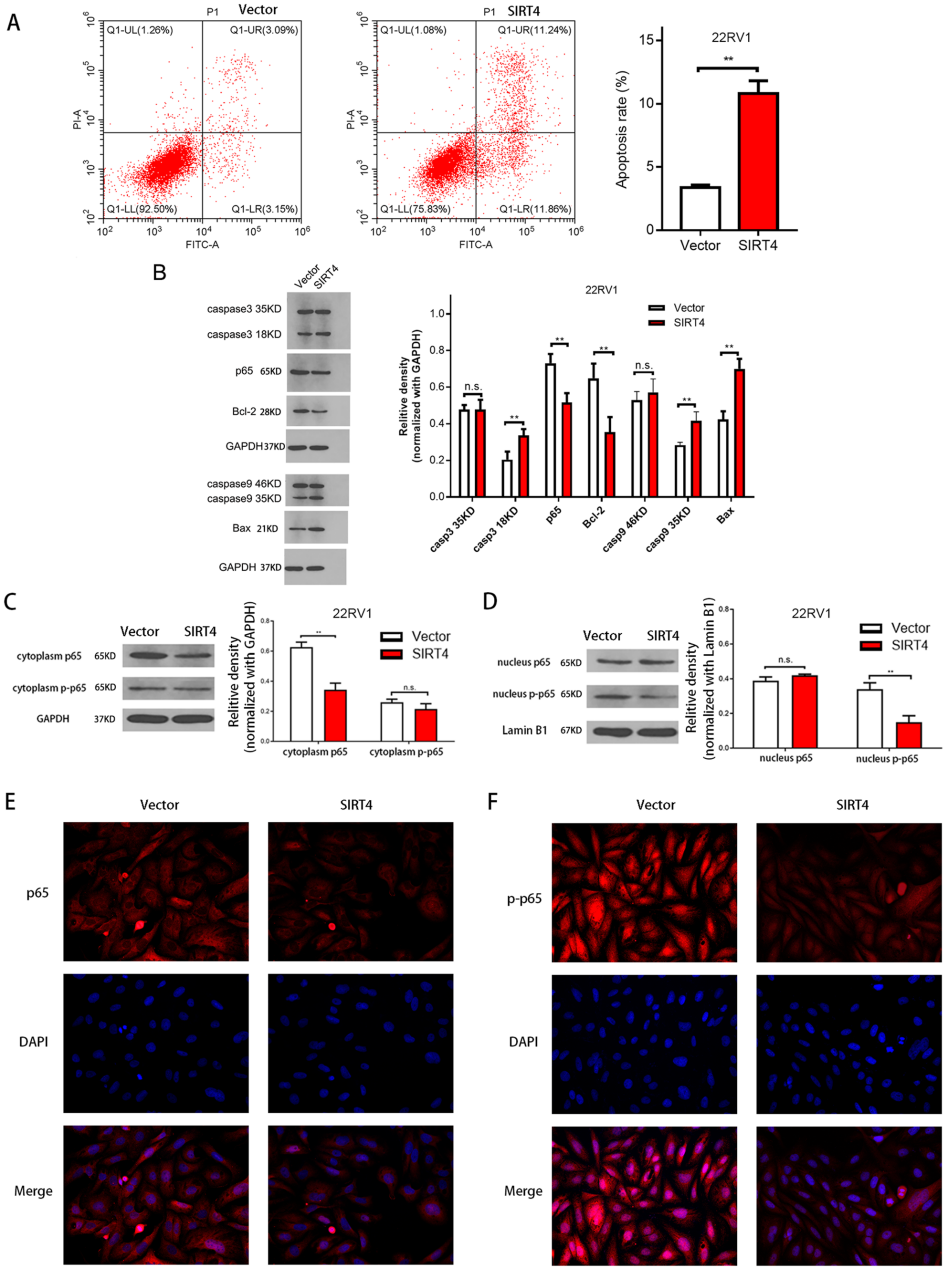
SIRT4 induces cell apoptosis through suppression of NF-κB activity by reducing the nuclear translocation of p65 in 22RV1 cells. (**A**) Vector and SIRT4 overexpression in 22RV1 cells is detected using FITC and PI staining with flow cytometry (C6; BD, Franklin Lakes, NJ,USA). (**B**) The relative density of caspase 3 and caspase 9proteins compared with GAPDH: density is analyzed and quantified using the Image J software (version 2.1.4.7; National Institutes of Health, Bethesda, MD, USA). Results are representative of three independent experiments and expressed as mean ± SD. The original blots are represented in Supplementary Fig. 2. (**C**) Effect of SIRT4 overexpression on p65 and p-p65 protein levels in the cytoplasm. The original blots are depicted in Supplementary Fig. 3. (**D**) Effect of overexpression of SIRT4 on p65 and p-p65 protein levels in the nucleus. The original blots are shown in Supplementary Fig. 3. (**E**) The effect of overexpression of SIRT4 on p65 levels in 22RV1 cells as observed by immunofluorescence. (**F**) The effect of overexpression of SIRT4 on p-p65 levels in 22RV1 cells as observed by immunofluorescence. *P < 0.05.**P < 0.01; NS, not significant.

Next, we explored the effects of SIRT4 on the proliferation of prostate cancer cells by inhibiting cell apoptosis. We found that apoptosis was significantly increased in 22RV1 cells with SIRT4 overexpression compared with that in the controls (Fig. 4A). To further investigate the molecular mechanism underlying SIRT4-induced apoptosis in 22RV1 cells, the expression of apoptotic regulatory proteins was detected by Western blotting following SIRT4 overexpression. SIRT4 overexpression resulted in a significant induction of caspase3 and caspase9 expressions (Fig. 4B), consistent with the flow cytometry findings. We also explored whether the NF-κB signaling pathway involved SIRT4-induced apoptosis in 22RV1. As shown in Fig. 4B, after Western blotting, SIRT4 led to a significant decrease in NF-κB/p65 phosphorylation. The protein levels of the target genes regulated by NF-κB transcription factors, such as Bcl-2 and Bax, were also examined. SIRT4 significantly downregulated the expression level of Bcl-2 (Fig. 4B). Moreover, a significant increase in the apoptotic protein Bax expression was observed after SIRT4 overexpression (Fig. 4B). In addition, SIRT4 overexpression reduced p65 levels in the cytoplasm (Fig. 4C) and p-p65 levels in the nucleus (Fig. 4D). A similar decline in p65 and p-p65 levels was noticed in 22RV1 cells with SIRT4 overexpression via immunofluorescence testing (Fig. 4E,F).

### SIRT4 regulates the proliferation, migration, and invasion capabilities of prostate cancer cells via inhibition of glutamine metabolism

**Figure 5:**
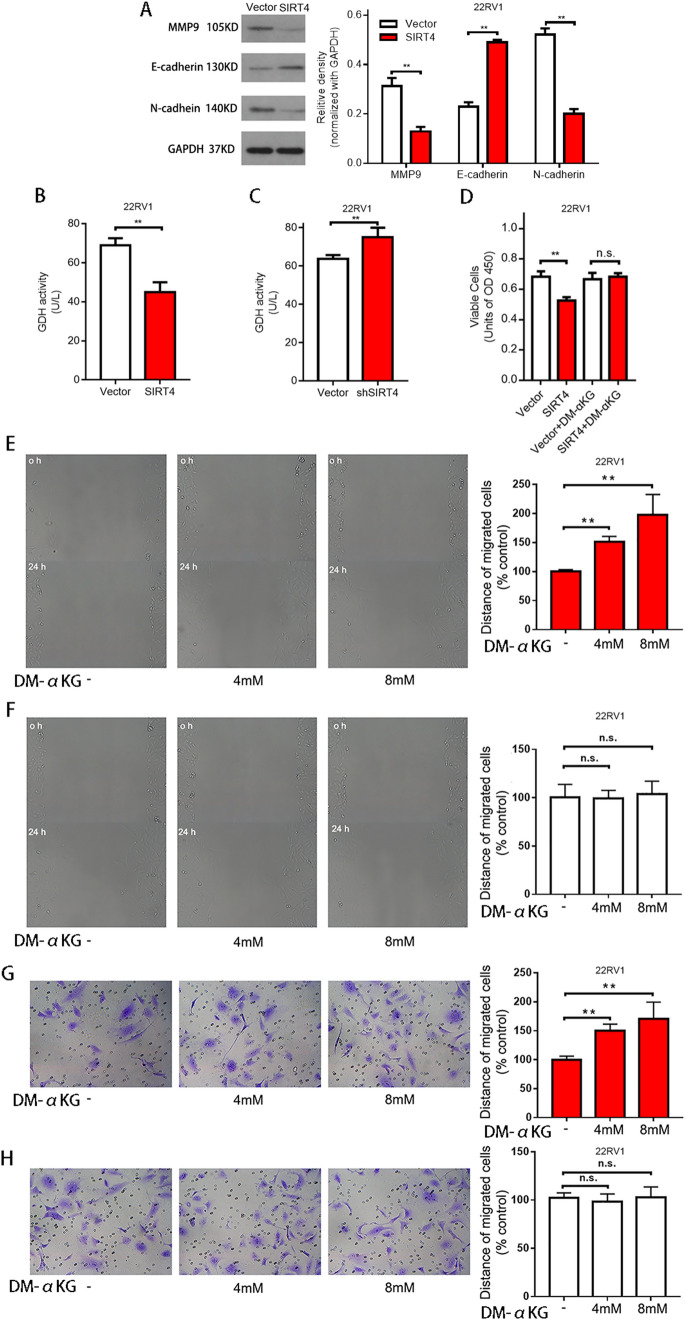
Regulation of cellular mobility by SIRT4 via suppression of glutamine metabolism. (**A**) The relative density of MMP9, E-cadherin, and N-cadherin proteins after comparison with GAPDH; the density is analyzed and quantified using the Image J software. The original blots are represented in Supplementary Fig. 4. (**B**) Glutamate dehydrogenase activity in control and 22RV1 cells overexpressing SIRT4. (**C**) Glutamate dehydrogenase activity in control and SIRT4 knockdown 22RV1 cells. (D) The proliferation activity of control vector-transfected and SIRT4-overexpression in 22RV1 cells in stand media or media supplemented with DM-αKG (8 mmol/L). Cell proliferative activity is measured at 72 h after seeding the cell. (**E**,**F**) The wound-healing assay with SIRT4-overexpressing 22RV1 (**E**) and control cells (**F**) treated with DM-αKG for 0 and 24 h, respectively. Representative images at the indicated time points are shown on the left (scale bar, 100 mm). (**G,H**) The cell invasion assay in SIRT4-overexpressing 22RV1 cells (**G**) and control cells (**H**) treated with DM-αKG after incubation for 0 and 24 h, respectively. Representative images at the indicated times are on the left (scale bar, 100 mm). Data are presented as mean ± SD of at least three independent experiments.**P < 0.01; NS, not significant.

To further elucidate the possible mechanisms underlying SIRT4-induced inhibition of invasion and migration, the levels of epithelial–mesenchymal transition (EMT)-related proteins were evaluated by Western blotting. SIRT4 overexpression notably increased E-cadherin expression significantly but reduced MMP9 and N-cadherin expressions (Fig. 5A). These results suggest that SIRT4 plays a crucial role in the invasion and migration of prostate cancer cells.

Accumulating evidence has shown that SIRT4 overexpression suppresses GDH enzymatic activity and limits the metabolism of glutamine and glutamate, thus generating adenosine triphosphates (ATPs)^4^. We found that GDH activity was significantly suppressed in 22RV1 cells after SIRT4 overexpression (Fig. 5B), whereas GDH activity was markedly increased after SIRT4 knockdown (Fig. 5C). Glutamine is converted into glutamic acid through glutaminase activity, and later, glutamate is translated to α-Ketoglutaric acid (α-KG) using GDH or a transaminase coupling reaction^17^. α-KG is a crucial metabolic product of glutamine metabolism. To determine whether SIRT4 inhibits the invasion and migration of prostate carcinoma cells by inhibiting glutamine metabolism, we treated 22RV1 cells with DM-αKG. We found reduced proliferation after revoking SIRT4 overexpression (Fig. 5D). Further, we observed that when DM-αKG was added to the medium, a significant increase was observed in the rate of wound healing in 22RV1 cells overexpressing SIRT4 (Fig. 5E); however, no change was observed in the wound healing rate in the negative control group on the addition of DM-αKG (Fig. 5F). Similarly, the invasion rate of 22RV1 cells overexpressing SIRT4 increased significantly when DM-ɑKG was added (Fig. 5G), but in the negative control cells, no significant change was observed (Fig. 5H). These results indicate that SIRT4 suppresses the proliferation, migration, and invasion capabilities of 22RV1 cells by inhibiting glutamine metabolism.

## Discussion

SIRT4 has been found to exhibit tumor suppressive role by virtue of its metabolic regulatory activity. However, there is still only limited information regarding the clinical significance and function of SIRT4 in tumorigenesis, and all agree studies suggest that it plays a role in inhibiting cancer genes. Recently, Jeong demonstrated that SIRT4 inhibits the formation of tumors by inhibiting glutamine metabolism^7^. Further, SIRT4 overexpression inhibits the proliferation ability of HeLa cells, and SIRT4 knockdown in MEF cells inhibits large tumor development in nude mice in vivo. Nevertheless, SIRT4-knockout mice spontaneously developed cancers of liver, lung, and breast. Furthermore, Csibi et al. showed that SIRT4 overexpression represses the proliferation ability of DU145 and DLD-1 cell lines^8^. Previously, SIRT4 expression in colorectal cancer has been demonstrated to lead to a decrease in unfavorable clinical outcomes associated with this tumor^10,11^. However, the relationship between SIRT4 and prostate cancer remains to be elucidated. In this study, using immunohistochemical analysis, we revealed that the expression of SIRT4 in prostate cancer tissues was significantly lower than that in adjacent non-neoplastic prostate tissues. Besides, we found that patients with low expression levels of SIRT4 exhibited a higher Gleason score. In our in vitro study, by overexpressing and inhibiting SIRT4 gene in prostate cancer cells, we demonstrated that SIRT4 could significantly inhibit cell proliferation, migration, invasion capabilities, and cell cycle of prostate cancer cells. These findings suggest that SIRT4 exhibits significant tumor-suppressing activities against prostate cancer cells. To the best of our knowledge, this study is the first study to reveal a significant association between SIRT4 protein expression levels and Gleason score in patients with prostate cancer.

Apoptosis loss can impact tumor initiation, progression, and metastasis^18^. Many studies have shown that the caspase family plays an essential role in apoptotic cell death. Caspase-9, a member of the executioner family, is vital in apoptotic incidents, whereas cleaved caspase-3 is a downstream protease of caspase-9 of the apoptotic pathway^19,20^. The nuclear factor-κB (NF-κB) family comprises five component subunits forming distinct transcriptionally active heterodimers or homodimers, including NF-κB1 and p65 (p105/p50), and displays anti-apoptotic effects^21^. The p65 subunit takes care of most of the roles of NF-κB^22^. In this study, SIRT4 overexpression elevated the protein levels of caspase-9 and cleaved caspase-3, and inhibited NF-κB activity by reducing the nuclear translocation of p65. These results indicated that SIRT4 inhibits the cellular proliferation of prostate cancer cells by inducing apoptosis.

One of the significant characteristics of tumors is the varied energy metabolism^23^. Tumor cells have a distinct metabolic pattern compared with normal cells; they frequently appear to increase glutamine metabolism and glucose metabolism to facilitate cell growth^24,25^. Therapeutics obstructing the metabolic pathway of tumor cells is a novel approach^26,27^. For example, using glucose inhibitors to obstruct the glucose metabolism pathway has been considered a useful tumor therapy method in studies^28^. However, tumor cells can also proliferate through the activation of glutamine metabolism when glucose metabolism is inhibited for cellular survival. Thus, glutamine metabolism is essential for tumor survival in a glucose-lacking environment^29^. Besides, recent research discovered that lack of glutamine could induce KRas-driven S-phase arrest in cancer cells, which is caused by inadequate nucleotide biosynthesis^30^; besides, these arrested cells are more sensitive to cytotoxic drugs such as rapamycin, paclitaxel, and capecitabine^31–33^, Consequently, blocking glutamine metabolism. Thus, inhibiting glutamine and glucose metabolism or administering synergistic chemotherapy drugs simultaneously is a promising approach in cancer therapy^34^. Previous studies have found that SIRT4 can inhibit glutamine metabolism^35^. In this study, we showed that overexpression of SIRT4 downregulated the proteins associated with migration and invasion and inhibited the proliferation, migration, and invasion capabilities of prostate cancer cells. These effects could be rescued by inhibiting glutamine metabolism. These results indicated the therapeutic potential of SIRT4 for targeting glutamine metabolism in prostate cancer, especially in combination with glucose metabolism inhibitors.

To the best of our knowledge, this is the first study to demonstrate the tumor suppressive activity of SIRT4 in prostate cancer cells through inhibition of glutamine metabolism and induction of cell apoptosis in vitro. Notably, a significant association of SIRT4 expression levels with Gleason score was also revealed in patients with prostate cancer. Thus, SIRT4 may serve as a potential novel therapeutic target for prostate cancer.

## Supplementary Information

Supplementary Information.

## Data Availability

The datasets used and/or analyzed during the current study are available from the corresponding author upon reasonable request.
