# Supplementary material for: SIRT4 functions as a tumor suppressor during prostate cancer by inducing apoptosis and inhibiting glutamine metabolism
Source: Sci Rep. 2022 Jul 16;12:12208. doi: 10.1038/s41598-022-16610-8 (PMC9288510; doi:10.1038/s41598-022-16610-8)

Supplementary Figure

Figure1. The original blots of Fig3B in manuscript.

Figure2. The original blots of Fig4B in manuscript.

Figure3. The original blots of Fig4C and Fig4D in manuscript.

Figure4. The original blots of Fig5A in manuscript.Figure1


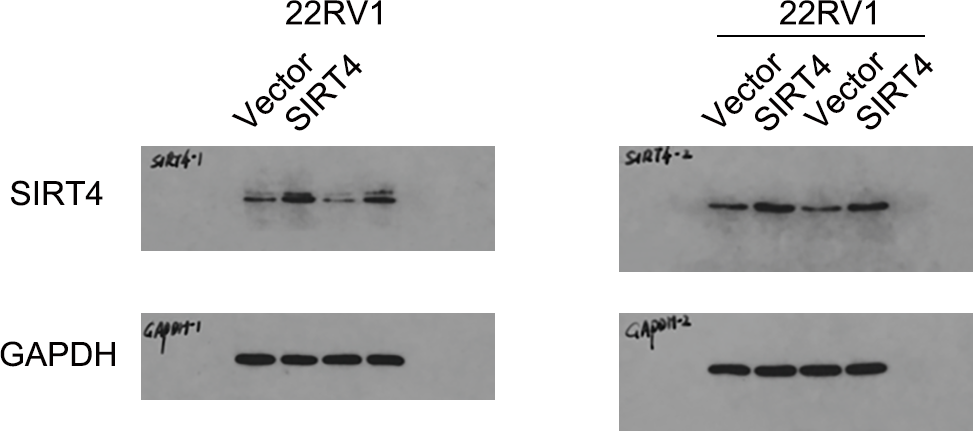


Figure2


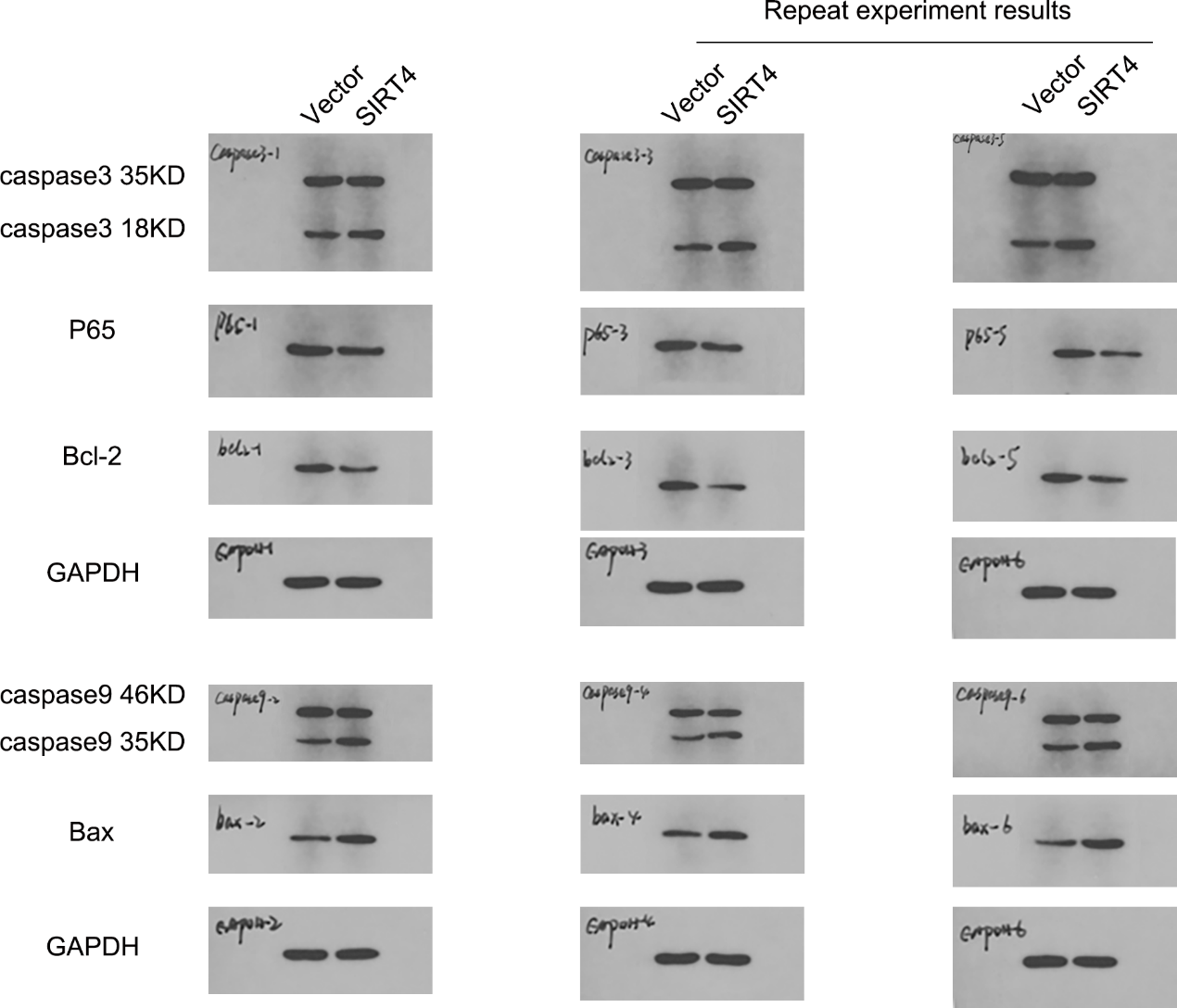


Figure3


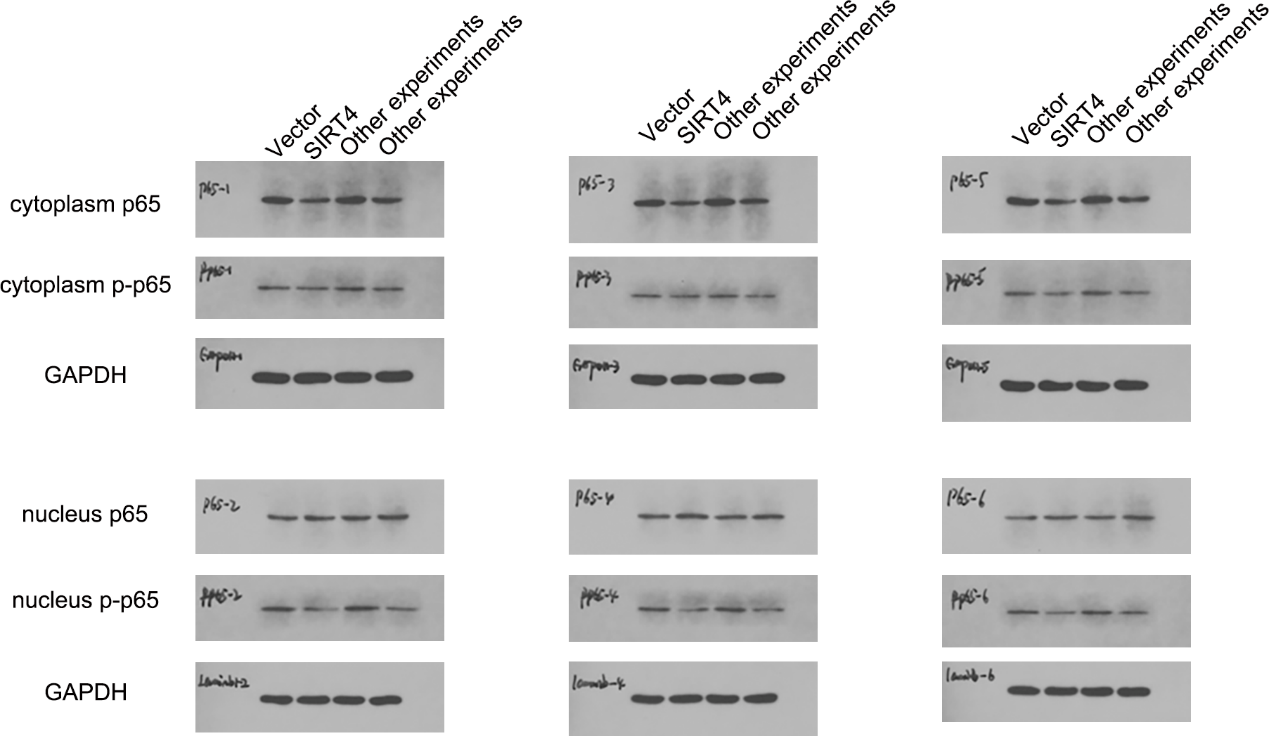


Figure4


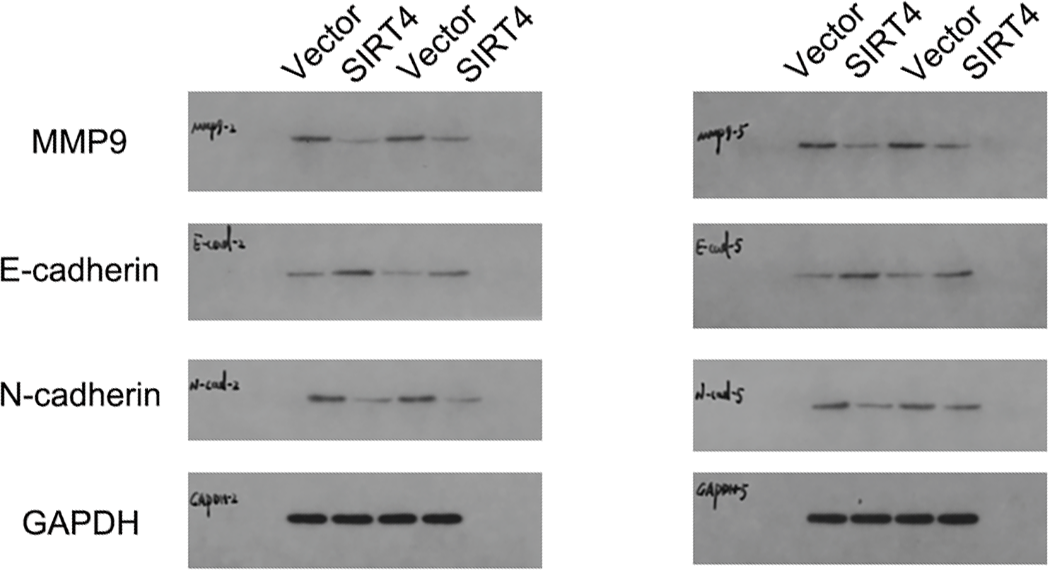

Supplement: Supplementary file 1 — Supplementary Information. [file 41598_2022_16610_MOESM1_ESM.docx]
